# Health-related quality of life one year after refractory cardiac arrest treated with conventional or extracorporeal CPR; a secondary analysis of the INCEPTION-trial

**DOI:** 10.1016/j.resplu.2024.100669

**Published:** 2024-05-30

**Authors:** Anina F. van de Koolwijk, Thijs S.R. Delnoij, Martje M. Suverein, Brigitte A.B. Essers, Renicus C. Hermanides, Luuk C. Otterspoor, Carlos V. Elzo Kraemer, Alexander P.J. Vlaar, Joris J. van der Heijden, Erik Scholten, Corstiaan A. den Uil, Dinis Dos Reis Miranda, Sakir Akin, Jesse de Metz, Iwan C.C. van der Horst, Bjorn Winkens, Jos G. Maessen, Roberto Lorusso, Marcel C.G. van de Poll, INCEPTION-investigators

**Affiliations:** a Department of Intensive Care Medicine, Maastricht University Medical Center+, Maastricht University, Maastricht, The Netherlands; b Department of Cardiology, Maastricht University Medical Centre+, Maastricht University, Maastricht, The Netherlands; c Department of Clinical Epidemiology and Medical Technical Assessment, Maastricht University Medical Center+, Maastricht University, Maastricht, The Netherlands; d Department of Cardiology, Isala Clinics, Zwolle, The Netherlands; e Department of Intensive Care, Catharina Hospital, Eindhoven, The Netherlands; f Department of Intensive Care, Leiden University Medical Center, Leiden University, Leiden, The Netherlands; g Department of Intensive Care, Amsterdam University Medical Center Location AMC, University, Amsterdam, Amsterdam, The Netherlands; h Department of Intensive Care, University Medical Center Utrecht, Utrecht University, Utrecht, The Netherlands; i Department of Intensive Care, St. Antonius Hospital, Nieuwegein, The Netherlands; j Department of Intensive Care, Erasmus Medical Center, Erasmus University, Rotterdam, The Netherlands; k Department of Cardiology, Erasmus Medical Center, Erasmus University, Rotterdam, The Netherlands; l Department of Intensive Care, Maasstad Hospital, Rotterdam, The Netherlands; m Department of Intensive Care, Haga Hospital, The Hague, The Netherlands; n Department of Intensive Care, OLVG, Amsterdam, The Netherlands; o Cardiovascular Research Institute Maastricht (CARIM), Maastricht University, Maastricht, The Netherlands; p Department of Methodology & Statistics, Maastricht University, Maastricht, The Netherlands; q Care and Public Health Research Institute, Maastricht University, Maastricht, The Netherlands; r Department of Cardiothoracic Surgery, Maastricht University Medical Center+, Maastricht University, Maastricht, The Netherlands; s School for Nutrition and Translational Research in Metabolism (NUTRIM), Maastricht University, Maastricht, The Netherlands

**Keywords:** Out-of-hospital cardiac arrest, Refractory arrest, Extracorporeal cardiopulmonary resuscitation, Health-related quality of life

## Abstract

**Background:**

Prospective, trial-based data comparing health-related quality of life (HRQoL) in patients surviving out-of-hospital cardiac arrest (OHCA) through extracorporeal cardiopulmonary resuscitation (ECPR) or conventional CPR (CCPR) are scarce. We aimed to determine HRQoL during 1-year after refractory OHCA in patients treated with ECPR and CCPR.

**Methods:**

We present a secondary analysis of the multicenter INCEPTION-trial, which studied the effectiveness of ECPR versus CCPR in patients with refractory OHCA. HRQoL was prospectively assessed using the EQ-5D-5L questionnaire. Poor HRQoL was pragmatically defined as an EQ-5D-5L health utility index (HUI) > 1 SD below the age-adjusted norm. We used mixed linear models to assess the difference in HRQoL over time and univariable analyses to assess factors potentially associated with poor HRQoL.

**Results:**

A total of 134 patients were enrolled, and hospital survival was 20% (27 patients). EQ-5D-5L data were available for 25 patients (5 ECPR and 20 CCPR). One year after OHCA, the estimated mean HUI was 0.73 (0.05) in all patients, 0.84 (0.12) in ECPR survivors, and 0.71 (0.05) in CCPR survivors (p-value 0.31). Eight (32%) survivors had a poor HRQoL. HRQoL was good in 17 (68%) patients, with 100% in ECPR survivors versus 60% in CCPR survivors (p-value 0.14).

**Conclusion:**

One year after refractory OHCA, 68% of the survivors had a good HRQoL. We found no statistically significant difference in HRQoL one year after OHCA in patients treated with ECPR compared to CCPR. However, numerical differences may be clinically relevant in favor of ECPR.

## Introduction

Out-of-hospital cardiac arrest (OHCA) is a life-threatening condition, annually affecting 67 to 170 per 100.000 inhabitants in European countries, with an average survival to hospital discharge varying from 0 to 18%^1^, ^2^, ^3^. Return of spontaneous circulation (ROSC) can be reached with early (bystander) basic life support and by the use of an automated external defibrillator.^4^ However, when defibrillation attempts are unsuccessful, the arrest is considered refractory, and the chances of ROSC and survival rapidly decline.^5^

In case of refractory OHCA, the use of extracorporeal cardiopulmonary resuscitation (ECPR) has increased in recent years. Survival rates up to 40% have been achieved with ECPR for refractory OHCA in well-organized and dedicated systems,^6^, ^7^, ^8^ although these high survival rates are not invariably reproduced.^9^, ^10^, ^11^ Two recent randomized trials that assessed the effectiveness of ECPR in patients with a presumed refractory OHCA found higher-than-expected survival with favorable neurologic outcome in patients allocated to CCPR.^7^, ^12^ These findings demonstrate that survival with favorable neurologic outcome after prolonged cardiac arrest due to ventricular arrhythmia is not uncommon, both through CCPR as well as through ECPR. While measures of neurologic function, such as cerebral performance category score, are frequently used to determine functional outcome following cardiac arrest, these scores do not necessarily reflect health-related quality of life (HRQoL).^13^ HRQoL plays a crucial role in determining whether interventions with significant costs, such as ECPR are cost-effective.^14^ Presently, data regarding HRQoL after OHCA and particularly after prolonged resuscitation is sparse, and little is known about factors affecting HRQoL after prolonged cardiac arrest.

In this paper, we present a secondary analysis of the multicenter randomized INCEPTION-trial that assessed the clinical effectiveness of ECPR in patients with a presumed refractory OHCA due to ventricular arrhythmia. The primary objective of this analysis was to compare HRQoL between patients surviving refractory arrest through CCPR and ECPR one year after OHCA. The secondary objective was to determine factors associated with poor HRQoL after prolonged OHCA.

## Methods

This study is a secondary analysis of a multicenter, randomized controlled trial conducted in the Netherlands. The trial protocol and primary outcomes have been published previously.^9^, ^15^ This multicenter trial took place in 10 centers with cardiac surgery programs. Inclusion took place from May 2017 to February 2021. The study protocol was approved by the ethics committee of Maastricht University (METC 162039). It was registered at clinicaltrials.gov (NCT03101787).

### Study population

Patients aged 18–70 years with witnessed refractory OHCA due to an initial ventricular arrhythmia (ventricular fibrillation, ventricular tachycardia, or shockable rhythm detected by automated external defibrillator), where bystander basic life support (BLS) was initiated, could be included. The arrest was considered refractory if there was no achievement of ROSC after 15 min despite advanced life support. Patients could be excluded based on their medical history (specified in original paper,^7^, ^12^ or in case of known contraindications for ECPR and expected time interval of more than 60 min between arrest and initiation of cannulation procedure. Patients whose actual time interval between arrest and initiation of cannulation exceeded 60 min after randomization were retained in the study.

### Data collection

In case of refractory arrest, intra-arrest transport to the hospital was initiated by emergency medical service. During the transport, patient information was sent to the receiving hospital. Patients could be included and randomized before arrival at the emergency department (ED). In case of stable ROSC before ECPR was initiated, ECPR was not applied.

Standard post-resuscitation care was delivered according to current guidelines and institutional protocols, which included temperature management and neurological assessment. Follow-up was performed 30 days, 3 months, 6 months, and 12 months after OHCA.

### Health-related quality of life

HRQoL was determined in survivors using the EQ-5D-5L questionnaire.^16^ This questionnaire records scores in 5 dimensions: mobility, self-care, usual activities, pain/discomfort, and anxiety/depression. Each dimension consists of five levels [1-5]: ranging from no problems [1] to extreme problems [5]. The scores on each level are translated to a single health utility index (HUI) using country-specific tariffs. EQ-5D-5L HUI is a score ranging from −0.59 to 1. A score of 1 presents perfect health, and a score below 0 represents a health state worse than death.^17^ Patients also gave an overall health score using the visual analog scale (VAS), ranging from 0 to 100, with 0 representing the worst possible health and 100 the best possible health.

### Definition of good and poor health-related quality of life

Since there are no cut-off values for good or poor HRQoL within the EQ-5D system, we applied a pragmatic definition of poor versus good HRQoL, based on the HUI at the last available follow-up moment. Poor HRQoL was defined as a HUI falling below one standard deviation of the age-matched general population (−1SD).^17^ Conversely, good HRQoL was defined as a HUI above −1SD.

### Statistical analysis

Numerical data were described as means (standard deviations), medians [interquartile range], and categorical data were summarized as numbers and percentages.

For the primary analysis (effect of ECPR versus CCPR on HRQoL), patients were divided into two groups according to the actual treatment received. Between-group differences in EQ-5D-5L HUI during the follow-up period were tested using mixed linear models with group, time, and group*time as fixed factors. With a random intercept on patient level to account for the correlation between repeated measures within a patient. For sensitivity analysis, data were analyzed using a marginal model for repeated measures with an unstructured covariance structure. Data are presented as estimated means (standard errors, SEs), as well as estimated differences (95% confidence intervals). If no significant difference between groups was found, overall estimated means were reported using a mixed linear model with only time as a factor.

For the secondary analysis, factors associated with HRQoL in survivors of refractory cardiac arrest, patients were grouped as good versus poor HRQoL, irrespective of the initial treatment. Factors that were potentially associated with HRQoL (age, pH, lactate, pCO_2_ and pO_2_ on arrival at ED, duration of resuscitation, length of ICU- and hospital admission^18^, ^19^ were tested using a Mann-Whitney *U* test for numerical variables and a Fisher’s exact test for categorical variables. Two-sided p-values are reported, whereas double 1-sided p-values were used for Fisher’s exact test. Statistical analyses were performed using SPSS (version 28.0.1.0). A p-value ≤ 0.05 was considered to indicate statistical significance.

## Results

**Fig. 1:**
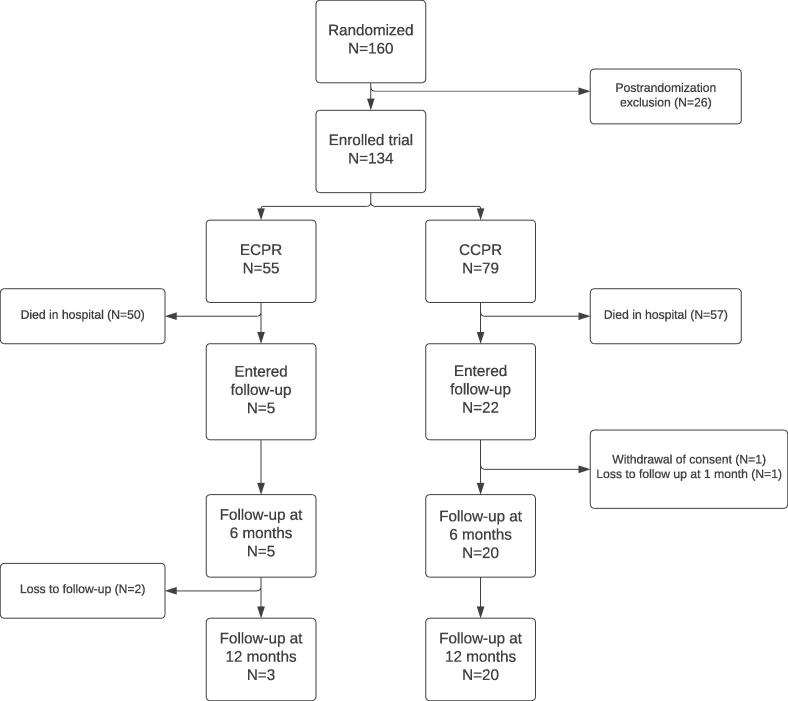
**Consort flow chart.** This secondary analysis followed an as-treated approach. A total of 134 patients were included in the trial. ECPR was initiated in 55 patients, resulting in 5 survivors, whereas 79 patients were treated with CCPR, resulting in 20 survivors. These also include patients randomized to ECPR who arrived at the hospital with ROSC. ECPR = extracorporeal cardiopulmonary resuscitation, CCPR = conventional cardiopulmonary resuscitation.

A total of 134 patients were included in the study, with a mean age of 55 (11) years. Ninety percent were male. Fifty-five patients were treated with ECPR. Of the entire study population, 82 patients were admitted to the ICU, of whom 54 died during the ICU stay. The median duration of hospitalization was 3 [1–13] days, and 27 patients survived to hospital discharge. One patient withdrew consent before the first follow-up moment, and one patient was lost to follow-up after that. All but one of the surviving patients had a CPC of 1 or 2. Twenty-five surviving patients were included in the current analysis (Fig. 1). Two patients were loss to follow-up between six and twelve months, due to unknown reasons. The baseline characteristics of the entire population and both treatment groups (as-treated) are described in Table 1. The last follow-up was 12 months in 23 patients and 6 months in 2 patients (both treated with ECPR).

**Table 1:** Baseline characteristics (as-treated analysis).

**Characteristic**	**All (N = 134)**	**ECPR (N = 55)**	**CCPR (N = 79)**
Age – years	55 (11)	54 (11)	55 (11)
Male sex − no.	120 (90)	51 (93)	69 (87)
Witnessed arrest – no.	131 (98)	55 (1 0 0)	76 (96)
CPR started ≤ 5 min after arrest – no.	131 (98)	55 (1 0 0)	75 (95)
Medical history – no.			
Acute coronary syndrome	20/116 (17)	9/46 (20)	11/70 (16)
Coronary artery disease	13/114 (11)	7/46 (15)	6/68 (9)
Chronic heart failure	6/116 (5)	4/47 (9)	2/69 (3)
Cerebrovascular accident	12/115 (10)	1/46 (2)	11/69 (16)
Diabetes mellitus	16/116 (14)	9/47 (19)	7/69 (10)
COPD / asthma	5/116 (4)	4/47 (9)	1/69 (1)
ROSC – no. of patients	38/134 (28)	25/55 (45)	42/79 (53)
Duration of arrest – min	45 (22)	75 (17)	44 (19)
Total number of defibrillations	7 [3–11]	7 [3–11]	7 [3–11]
pH at arrival ED*	6.84[6.79–6.99]	6.87 [6.80–7.01]	6.82[6.75–6.99]
pCO2 at arrival ED	9.7 [6.6–12.9]	9.0 [6.7–11.8]	9.9 [6.2–13.3]
pO2 at arrival ED	6.1 [2.0–10.3]	7.2 [2.1–12.0]	5.4 [2.0–10.2]
Lactate at arrival ED**	13.5 [10.5–16.3]	13.8 [10.8–17.0]	13.2 [9.9–16.0]
ICU stay			
No. of patients	82 (61)	48 (87)	34 (43)
Median duration of stay – days	2 [1–4]	1 [1–5]	2 [1–4]
Hospitalization			
No. Of patients	78 (58)	45 (82)	33 (42)
Median duration of stay – days	3 [1–13]	1 [1–4]	12 [3–18]
Death after ICU admission – no.***	54 (66)	40 (83)	9 (26)
Survived to ICU discharge – no.	29 (22)	5 (9)	24 (30)
Survived to hospital discharge – no.	27 (20)	5 (9)	22 (28)
Neurological function at 30 days****			
CPC 1	20 (15)	3 (5)	17 (22)
CPC2	4 (3)	2 (4)	2 (3)
CPC3	1 (1)	0 (0)	1 (1)
CPC4	0 (0)	0 (0)	0 (0)
CPC 5	107 (80)	50 (91)	57 (72)

Data are mean (SD), median [IQR], or number of patients (%). Percentages are rounded up and may not add up to 100%.

BLS = basis life support, COPD = chronic obstructive pulmonary disease, ECPR = extracorporeal cardiopulmonary resuscitation, CCPR = conventional cardiopulmonary resuscitation, ROSC = return of spontaneous circulation, ICU = intensive care unit, CPC = cerebral performance category.

* Data of 118 patients.

** Data of 106 patients.

*** Percentage of people who were admitted to ICU.

**** One patient withdrew informed consent before 30 days follow-up, and one patient was not assessed by a neurologist at 30 days follow-up, both in the CCPR group.

### Health-related quality of life after ECPR versus CCPR

**Fig. 2:**
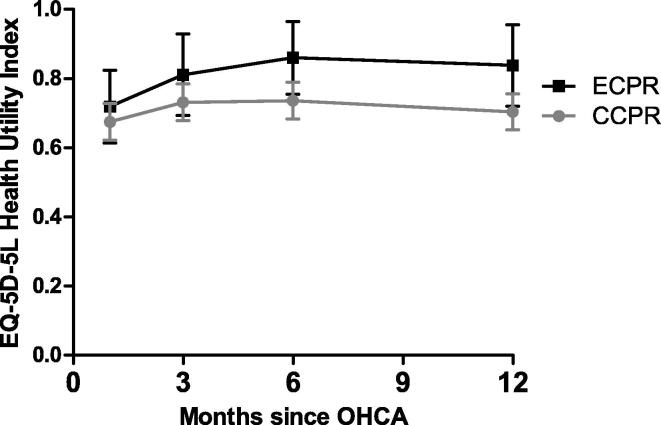
**Estimated mean Health Utility Index over time in survivors of refractory OHCA surviving through ECPR (n = 5) versus CCPR (n = 20).** As-treated analysis was performed using linear mixed model with random intercept. There was no significant interaction between group and time (p = 0.763); data are presented as estimated mean (95% CI).

The longitudinal course of the EQ-5D-5L HUI during the first year in patients treated with ECPR versus CCPR is depicted in Fig. 2. There was no statistically significant difference in longitudinal trend between patients who received either ECPR or CCPR (p-value for interaction between group and time 0.763). At 12 months after OHCA, the overall estimated mean (SE) EQ-5D-5L HUI was 0.73 (0.05). In patients who received ECPR, the estimated mean (SE) HUI at 12 months was 0.84 (0.12), and 0.71 (0.05) for patients treated with CCPR. The estimated difference of 0.13 (95%CI −0.12–0.31) was not statistically significant (p-value 0.31). The same applies to the differences at 30 days, 3 months, and 6 months (p-value 0.74, 0.55, 0.30, respectively). Similar results were found in the sensitivity analysis (marginal model with unstructured covariance structure, data not shown). Utility scores per dimension of the EQ-5D-5L questionnaire are shown in Table 2. There were no significant differences in any HRQoL dimensions between patients who received ECPR and those who received CCPR. The distributions of the domain scores of the EQ-5D-5L questionnaire are shown in Figure S1 (supplements).

**Table 2:** Estimated means of EQ-5D-5L scores and CPC scores in survivors of refractory OHCA treated with ECPR versus CCPR at 12 months follow-up.

	**All (N = 25)**	**ECPR (N = 5)**	**CCPR (N = 20)**	**Estimated difference (95% CI)**	**P-value**
**CPC-score**					
CPC 1	20/23 (87)*	3/3 (1 0 0)*	17/20 (85)		
CPC 2	2/23 (9)	0/3 (0)	2/20 (10)		0.33
CPC 3	1/23 (4)	0/3 (0)	1/20 (5)		
**Dimension of EQ-5D-5L**					
Mobility	1.52 (0.17)	1.48 (0.44)	1.55 (0.19)	−0.66 (−1.02–0.89)	0.89
Self-care	1.33 (0.13)	1.62 (0.35)	1.30 (1.45)	0.35 (−0.44–1.08)	0.39
Activity	2.09 (0.28)	1.41 (0.72)	2.20 (0.32)	−0.79 (−2.38–0.79)	0.32
Pain	1.93 (0.21)	1.48 (0.57)	2.00 (0.34)	−0.52 (−1.75–0.71)	0.40
Anxiety	1.73 (0.20)	1.38 (0.52)	1.80 (0.22)	−0.42 (−1.55–0.71)	0.46
Rated health (VAS)	70.6 (4.0)	70.4 (10.2)	71.0 (4.3)	−0.6 (–22.8 – 21.7)	0.96
HUI	0.73 (0.05)	0.84 (0.12)	0.71 (0.05)	0.13 (−0.12–0.31)	0.31

Cerebral performance category scores are presented as number of patients (%) and analyzed using the Fisher’s exact test. Data on EQ-5D-5L are presented as an estimated mean with standard error using mixed linear models with random intercept. Estimated difference is shown with 95% confidence intervals (CI).

CPC = cerebral performance category, ECPR = extracorporeal cardiopulmonary resuscitation,

CCPR = conventional cardiopulmonary resuscitation, VAS = visual analog scale, HUI = health utility index.

* CPC score at 12 months was missing in two patients.

### Factors associated with poor health-related quality of life

Poor HRQoL, defined as a HUI at last follow-up more than −1SD below the age-matched general population, was found in 8/25 patients surviving refractory cardiac arrest.

Age, sex, number of defibrillations, duration of cardiac arrest, or metabolic parameters, such as pH or lactate, did not differ significantly between patients with a good HRQoL and patients with a poor HRQoL (Table 3). Interestingly, a good HRQoL was found in 5/5 patients treated with ECPR versus 12/20 in patients treated with CCPR. However, this difference did not reach statistical significance (p-value 0.23). There were also no statistically significant differences in the duration of ICU admission or duration of hospitalization between patients with a good HRQoL and patients with a poor HRQoL.

**Table 3:** Factors associated with health-related quality of life in refractory OHCA.

**Parameter**	**Good (N = 17)**	**Poor (N = 8)**	**P-value**
Age − years	57 [44–63]	55 [38–64]	0.79
Sex			
Male	16 (70)	7 (30)	1.00
Female	1 (50)	1 (50)	
Total number of defibrillations	9 [6–13]	9 [7–11]	0.43
Duration of the arrest – min	35 [23–65]	49 [30–54]	0.68
pH at arrival ED*	7.02 [6.87–7.17]	7.04 [6.88–7.14]	1.00
pCO2 at arrival ED*	8.4 [6.1–11.4]	7.9 [6.8–9.1]	0.24
pO2 at arrival ED*	11.9 [7.1–35.8]	10.3 [7.1–32.1]	0.39
Lactate at arrival ED**	11.3 [7.5–15.2]	11.3 [6.1–14.7]	0.89
Treatment			
ECPR	5 (1 0 0)	0 (0)	0.23
CCPR	12 (60)	8 (40)	
(Intermittent) ROSC during resuscitation			
Yes	15 (65)	8 (35)	0.91
No	2 (1 0 0)	0 (0)	
Median ICU stay – days	4 [2–5]	3 [2–14]	0.81
Median hospital stay – days**	16 [11–25]	19 [10–36]	0.54

Numerical data are presented as median [IQR] and compared using Mann Whitney *U* test. Categorical data are presented as no. (%) and compared using Fisher’s exact test.

ED = emergency department, ECPR = extracorporeal cardiopulmonary resuscitation, CCPR = conventional cardiopulmonary resuscitation, ROSC = return of spontaneous circulation, ICU = intensive care unit.

* Data of 24 patients, in kPa.

** Data of 21 patients.

Estimated mean disutility scores on the 5 domains of the EQ-5D-5L questionnaire at 12 months after refractory cardiac arrest in patients with a good HRQoL and patients with a poor HRQoL are presented in Table 4. In patients categorized as having poor HRQoL, the estimated mean EQ-5D-5L HUI was 0.44 (0.06). Patients with good HRQoL had an estimated mean of 0.87 (0.05). Patients with a poor HRQoL particularly reported problems in the domains of usual activities and pain but to a lesser extent in the domain of self-care Figure S2. (supplements).

**Table 4:** Estimated means of EQ-5D-5L and CPC scores in survivors of refractory OHCA with good and poor health-related quality of life.

	**Good (N = 17)**	**Poor (N = 8)**	**Estimated difference (95% CI)**	**P-value**
**CPC score**				
CPC 1	14/15 (93)*	6/8 (75)		
CPC 2	1/15 (7)	1/8 (12.5)		0.62
CPC 3	0/15 (0)	1/8 (12.5)		
**Dimension of EQ-5D-5L**				
Mobility	1.22 (0.18)	2.13 (0.25)	−0.90 (−1.50- −0.29)	<0.01
Selfcare	1.26 (1.53)	1.50 (0.21)	−0.24 (−0.77––0.28)	0.36
Activity	1.39 (0.30)	3.50 (0.41)	−2.11 (−3.14- −1.09)	<0.01
Pain	1.44 (0.23)	2.88 (0.32)	−1.44 (−2.22– −0.65)	<0.01
Anxiety	1.33 (0.23)	2.50 (0.33)	−1.17(−1.97- −0.37)	<0.01
Rated health (VAS score)	76.3 (4.5)	59.6 (6.3)	16.7(1.1––32.2)	0.04
HUI	0.87 (0.05)	0.44 (0.06)	0.43 (0.27––0.59)	<0.01

Poor functional outcome is defined as an EQ-5D-5L Health Utility Index more than 1 standard deviation below the age-matched general population.

Cerebral performance category scores are presented as number of patients (%) and using the Fisher’s exact test. Data on EQ-5D5L are presented as estimated mean with standard error using mixed linear models with random intercept.

CPC = cerebral performance category, VAS = visual analogue scale, HUI = health utility index.

* CPC score at 12 months was missing in two patients.

### Cerebral performance score

Twelve months after refractory OHCA, 22/23 of all surviving patients who completed the follow-up had a CPC-score of 1 or 2, which would generally be classified as a good outcome. All patients treated with ECPR had a CPC-score of 1 at 12 months. Of the patients treated with CCPR, 17/20 had a CPC score of 1 at 12 months. The difference between both groups is not statistically significant (Table 2, p-value 0.33). Six of 20 patients with a CPC score of 1 were deemed to have a poor HRQoL (Table 4).

## Discussion

This secondary analysis of the multicenter randomized INCEPTION-trial found no statistically significant difference in HRQoL one year after refractory OHCA between patients surviving refractory arrest through CCPR or ECPR. Throughout the study population, 96% of the surviving patients had a CPC score of 1 or 2, indicating a favorable neurological outcome.

The use of the CPC score in cardiac arrest research is increasingly scrutinized as it may not reflect functional outcome.^20^ In general, CPC scores of 1 and 2 are classified as good outcome after resuscitation. While these patients are survivors with good neurological function, this does not necessary mean they experience a good quality of life or good functional outcome. Larsson et al. showed that patients with CPC score of 1, had a better HRQoL than patients with a CPC-score of 2.^13^ Our research showed that one year after refractory OHCA, most patients had a CPC score of 1. While this is typically categorized as a favorable outcome, it is important to note that not all patients had a good HRQoL. To this end, HRQoL instruments, such as the EQ-5D-5L questionnaire, may be a better measure for good outcome. This questionnaire is designed to calculate a health utility, to be used in cost-effectiveness studies. The INCEPTION trial showed that in case of pragmatic implementation of ECPR for OHCA, the probability of cost-effectiveness is low.^14^ While this is important, it is equally crucial to focus specifically of HRQoL. When using the HUI for cost-effectiveness the utility score is calculated based on survivors and non-survivors. The current analysis provides information regarding different aspects of HRQoL of survivors and gives insight in possible problems of survivors. Such as in which domain the most problems occur. These insights can help us arrange the post-OHCA care effectively. However, these instruments do not provide clear cut-off values indicative of poor HRQoL. Therefore, we pragmatically defined cut-off values for poor HRQoL based on the age-adjusted means and standard deviations of the EQ-5D-5L HUI in a reference population. We found that one-third of the surviving patients had a HUI at least one standard deviation below the mean of the age-adjusted norm population. When considering such a deviation indicative of a poor HRQoL, all patients treated with ECPR had a good HRQoL, with a mean HUI of 0.84 (0.12) and a VAS score of 70.4 (10.2) after 12 months, compared to 60% of the patients treated with CCPR with a mean HUI of 0.71 (0.05) and a VAS score of 71.0 (4.3). The observed difference in HRQoL between ECPR survivors and CCPR survivors substantially exceeds the minimal clinically important difference from a patient perspective.^20^, ^21^ However, no conclusions can be drawn due to the small sample size.

In general, the reported quality of life in OHCA survivors is relatively good. Different studies show a HUI ranging from 0.78 to 0.85 after cardiac arrest and a rated health VAS score from 71 to 80,^21^, ^22^, ^23^, ^24^ which is comparable to the general population. The overall mean HUI in the present study was lower, with an estimated mean HUI of 0.73 (0.05) and a rated health VAS of 70.6 (4.0). This difference may be explained by the fact that the mean (SD) arrest duration in this cohort was 45 (22) min, which is much longer than the typical duration of a successful resuscitation. However, in this study, we found no significant association between the duration of resuscitation and HRQoL. This seems to align with results from Chai et al., who also found no significant association between the duration of resuscitation and long-term functional outcome (defined as survival without any personal care required) in a large cohort of OHCA survivors.^25^ It should be noted that the median duration of resuscitation between that study and the present one differs substantially, which may preclude the comparability of both studies. Moreover, problems with self-care, which were used by Chai et al. to define functional outcome were less prominent than problems on other HRQoL domains in patients with the lowest HUI scores.

Previous research on HRQoL after refractory OHCA treated with ECPR is limited, but more data have been published as ECPR is increasingly applied. The reported HRQoL after ECPR varies amongst studies. The most recent publication, a long-term follow-up after the Prague OHCA trial, comparing HRQoL after ECPR and CCPR reported no significant differences between both groups.^26^ The EQ-VAS value was 71 in ECPR-based group and 76 in CCPR group. This is comparable to the results of our study. Another recent study of Gregers and colleagues showed a median HUI of 0.73 (0.67–0.86) in patients treated with ECPR in case of refractory OHCA.^27^ This is a substantially lower than in our study. While lactate and pH levels are comparable, the low flow time is longer. The median duration of low flow time was 86 min. Another possible explanation is that not all patients had a witnessed arrest or initial shockable rhythm. Hodgson and colleagues published a multicenter study on HRQoL after extracorporeal membrane oxygenation (ECMO) for different indications: respiratory failure, cardiogenic shock, and ECPR.^28^ They included almost all cases of ECMO use in Australia; in the case of ECPR, this included patients suffering from in-hospital cardiac arrest as well as OHCA. HRQoL after 6 months of follow-up was compared with retrospectively assessed pre-existent functioning. The mean HUI of patients treated with ECPR was 0.80, with a VAS health score of 75. There was no statistically significant change in overall HRQoL, but 25% of the survivors reported new problems in the domains of mobility and pain. In our data, we found that patients with poor HRQoL particularly reported problems with pain and daily activities. In addition, Hodgson et al. found that HRQoL was similar in veno-arterial ECMO, veno-venous ECMO, and ECPR survivors. Using a comparable approach, Oude Lansink et al. studied the HRQoL after ECMO for different indications in a nationwide Dutch registry study.^29^ They found a mean HUI of 0.64 with a VAS health score of 64 in ECPR survivors, which is lower than we found in our present data. In fact, the HUI observed by Oude Lansink et al. was more than 1 standard deviation below the mean HUI in any age category, indicating an overall poor HRQoL in this particular cohort. Furthermore, they found that HRQoL after ECPR was substantially lower than HRQoL values after ECMO for respiratory or cardiac failure. Interestingly, the HRQoL of our ECPR cohort is comparable with HRQoL after ECMO for respiratory or cardiac failure in their report. An explanation for the discrepancies between HRQoL after ECPR remains elusive since details on patient and cardiac arrest characteristics reported by Oude Lansink et al. are limited. The strict and prospective selection of patients with the best chances of good functional survival (acute onset, witnessed arrest, good premorbid performance) may have contributed to better outcome in our cohort. Spangenberg et al. observed that HRQoL in OHCA and IHCA patients treated with ECPR was lower than in the general population and lower than in veno-venous ECMO survivors but comparable with HRQoL of veno-arterial ECMO survivors and patients with chronic renal failure.^30^

### Strengths and limitations

This is a secondary analysis of a multicenter, prospective randomized controlled trial, owing to the strict selection criteria and prospective design, selection bias is limited. The selection criteria are in line with current guidelines on ECPR. In addition, the follow-up schedule was preplanned and predefined, resulting in a high standardization. Complete loss to follow-up was limited to two patients. By using mixed linear models, all available data were used, also the observed data from the two patients dropping out before the final follow-up. Due to this dropout, the CPC score at 12 months could only be assessed for 3 out of 5 EPCR survivors. However, at 6 months, all 5 ECPR survivors had a CPC-score of 1. Another strength is the follow-up duration of one year. After cardiac arrest, it takes time to recover and rehabilitate; a shorter follow-up could result in a lower quality of life.

A limitation is the small sample size. There was a numerical, clinically relevant difference between patients treated with both treatments, but this difference did not reach statistical significance. Conclusions on the effect of ECPR versus CCPR on functional outcome should be drawn with care. We measured HRQoL using the EQ-5D-5L, which is a widely used tool for determining HRQoL. But it is important to note that this is not a specifically tool for cardiac arrest survivors, meaning specific problems after cardiac arrest such as fatigue, speech and language difficulties and cognition may remain unnoticed. Another limitation is the predominantly male sample size, conclusions regarding females should be drawn with care. Given that OHCA predominantly affects males, the findings of the current study may accurately reflect the demographics of patients surviving OHCA.^3^ Since the original study used prehospital randomization, many patients allocated to ECPR regained ROSC before hospital arrival. Since patients who regain ROSC before hospital arrival have the best prognosis, an intention-to-treat analysis may overestimate the clinical effectiveness of ECPR. Therefore, this secondary analysis was performed using an as-treated approach.

## Conclusion

A good HRQoL can be achieved, when patients survive an initial refractory OHCA, either through CCPR or ECPR. We found no statistically significant difference in HRQoL after ECPR or CCPR, however the numerical differences may be clinically relevant in favor of ECPR. We found no factors statistically significant associated with poor HRQoL. Due to small sample size conclusions should be drawn with care.

## Funding

This paper represents independent research, financially supported by Getinge and ZonMw, to conduct the original trial.

## CRediT authorship contribution statement

**Anina F. van de Koolwijk:** Writing – original draft, Methodology, Investigation, Formal analysis. **Thijs S.R. Delnoij:** Writing – review & editing, Validation, Data curation, Conceptualization. **Martje M. Suverein:** Writing – review & editing, Validation, Investigation, Data curation, Conceptualization. **Brigitte A.B. Essers:** Writing – review & editing, Validation, Methodology. **Renicus C. Hermanides:** Writing – review & editing, Validation, Resources, Investigation. **Luuk C. Otterspoor:** Writing – review & editing, Validation, Resources, Investigation. **Carlos V. Elzo Kraemer:** Writing – review & editing, Validation, Resources, Investigation. **Alexander P.J. Vlaar:** Writing – review & editing, Validation, Resources, Investigation. **Joris J. van der Heijden:** Writing – review & editing, Validation, Resources, Investigation. **Erik Scholten:** Writing – review & editing, Validation, Resources, Investigation. **Corstiaan A. den Uil:** Writing – review & editing, Validation, Resources, Investigation. **Dinis Dos Reis Miranda:** Writing – review & editing, Validation, Resources. **Sakir Akin:** Writing – review & editing, Validation, Resources, Investigation. **Jesse de Metz:** Writing – review & editing, Validation, Resources, Investigation. **Iwan C.C. van der Horst:** Writing – review & editing, Validation, Supervision, Methodology. **Bjorn Winkens:** Writing – review & editing, Validation, Methodology, Formal analysis. **Jos G. Maessen:** Writing – review & editing, Validation, Supervision, Resources, Methodology, Conceptualization. **Roberto Lorusso:** Writing – review & editing, Validation, Supervision, Resources, Methodology, Conceptualization. **Marcel C.G. van de Poll:** Writing – review & editing, Writing – original draft, Validation, Supervision, Resources, Methodology, Funding acquisition, Formal analysis, Conceptualization.

## Declaration of competing interest

The authors declare that they have no known competing financial interests or personal relationships that could have appeared to influence the work reported in this paper.
