# Supplementary material for: Health-related quality of life one year after refractory cardiac arrest treated with conventional or extracorporeal CPR; a secondary analysis of the INCEPTION-trial
Source: Resusc Plus. 2024 May 30;19:100669. doi: 10.1016/j.resplu.2024.100669 (PMC11170473; doi:10.1016/j.resplu.2024.100669)
Supplement: Supplementary Data 2 [file mmc2.pdf]

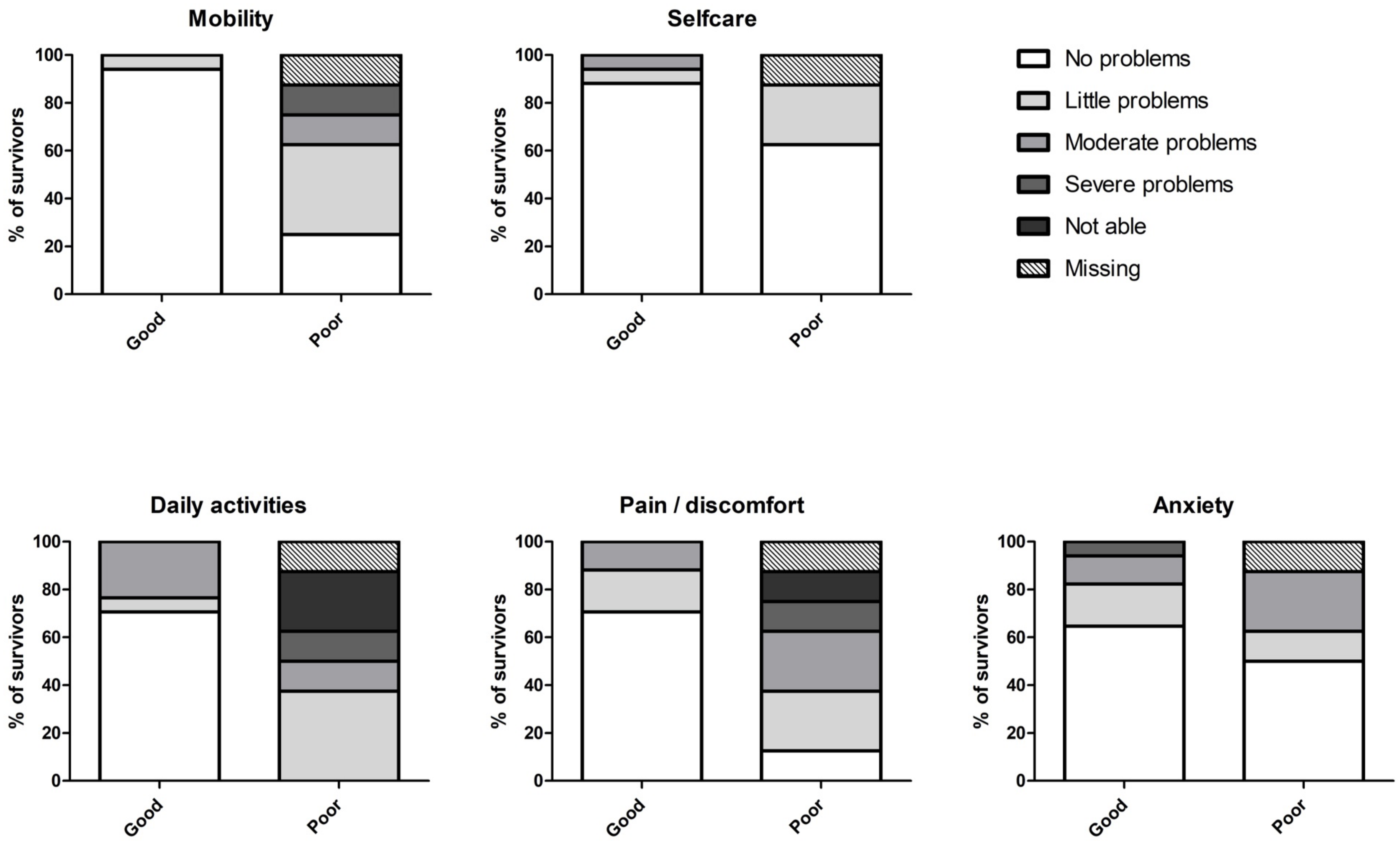

S2. EQ-5D-5L percentage of patients per domain, poor versus good outcome

Percentage of patients per domain of the EQ-5D-5L questionnaire at 6 months after OHCA. Good survival contains 17 patients, poor survival contains 8 patients.
